# A-910823, a squalene-based emulsion adjuvant, induces T follicular helper cells and humoral immune responses *via* α-tocopherol component

**DOI:** 10.3389/fimmu.2023.1116238

**Published:** 2023-02-20

**Authors:** Yuya Yoshioka, Kouji Kobiyama, Tomoya Hayashi, Motoyasu Onishi, Yosuke Yanagida, Takayuki Nakagawa, Masayuki Hashimoto, Anri Nishinaka, Jun Hirose, Yoshiji Asaoka, Minako Tajiri, Atsushi Hayata, Satoru Ishida, Shinya Omoto, Morio Nagira, Ken J. Ishii

**Affiliations:** ^1^ Laboratory for Bio-Drug Discovery, Shionogi & Co., Ltd., Osaka, Japan; ^2^ Division of Vaccine Science, Department of Microbiology and Immunology, The Institute of Medical Science, The University of Tokyo, Tokyo, Japan; ^3^ International Vaccine Design Center, The Institute of Medical Science, The University of Tokyo, Tokyo, Japan; ^4^ Formulation R&D Laboratory, Shionogi & Co., Ltd., Osaka, Japan; ^5^ Laboratory for Drug Discovery and Development, Shionogi & Co., Ltd., Osaka, Japan; ^6^ Laboratory for Bio-Modality Research, Shionogi & Co., Osaka, Japan; ^7^ Vaccine and Adjuvant Research Center (CVAR), National Institute of Biomedical Innovation, Health and Nutrition (NIBIOHN), Osaka, Japan; ^8^ Laboratory of Vaccine Science, Immunology Frontier Research Center, Osaka University, Osaka, Japan

**Keywords:** adjuvant, A-910823, vaccine, S-268019-b, Tfh, mouse, immune response, severe acute respiratory syndrome coronavirus 2

## Abstract

**Background:**

Adjuvants are chemical or biological materials that enhance the efficacy of vaccines. A-910823 is a squalene-based emulsion adjuvant used for S-268019-b, a novel vaccine against severe acute respiratory syndrome coronavirus 2 (SARS-CoV-2) that is currently in clinical development. Published evidence has demonstrated that A-910823 can enhance the induction of neutralizing antibodies against SARS-CoV-2 in humans and animal models. However, the characteristics and mechanisms of the immune responses induced by A-910823 are not yet known.

**Methods and Results:**

To characterize A-910823, we compared the adaptive immune response profile enhanced by A-910823 with that of other adjuvants (AddaVax, QS21, aluminum salt-based adjuvants, and empty lipid nanoparticle [eLNP]) in a murine model. Compared with other adjuvants, A-910823 enhanced humoral immune responses to an equal or greater extent following potent T follicular helper (Tfh) and germinal center B (GCB) cell induction, without inducing a strong systemic inflammatory cytokine response. Furthermore, S-268019-b containing A-910823 adjuvant produced similar results even when given as a booster dose following primary administration of a lipid nanoparticle-encapsulated messenger RNA (mRNA-LNP) vaccine. Preparation of modified A-910823 adjuvants to identify which components of A-910823 play a role in driving the adjuvant effect and detailed evaluation of the immunological characteristics induced by each adjuvant showed that the induction of humoral immunity and Tfh and GCB cell induction in A-910823 were dependent on α-tocopherol. Finally, we revealed that the recruitment of inflammatory cells to the draining lymph nodes and induction of serum cytokines and chemokines by A-910823 were also dependent on the α-tocopherol component.

**Conclusions:**

This study demonstrates that the novel adjuvant A-910823 is capable of robust Tfh cell induction and humoral immune responses, even when given as a booster dose. The findings also emphasize that α-tocopherol drives the potent Tfh-inducing adjuvant function of A-910823. Overall, our data provide key information that may inform the future production of improved adjuvants.

## 1 Introduction

Adjuvants are chemical or biological materials that are added to a vaccine to stimulate and enhance the magnitude and durability of the vaccine antigen-specific immune response (1, 2). Vaccine adjuvants can be broadly classified into several groups, including mineral salts, emulsions, biological molecules, and synthetic materials (2). Adjuvants can directly or indirectly stimulate the innate arm of the immune system and can also modulate the adaptive arm of the host defense system (1). Characteristic mechanisms employed by adjuvants to elicit immune responses include sustained release of antigen at the site of injection (depot effect); up-regulation of cytokines and chemokines; cellular recruitment at the site of injection; increased antigen uptake and presentation to antigen presenting cells (APCs); activation and maturation of APCs and migration to the draining lymph nodes; and activation of inflammasomes, including antibody induction (2, 3).

Aluminum salt-based adjuvants (alum) were the first to be used in licensed human vaccines and are still the most widely used adjuvant (4). Although they have a wide-spectrum ability to strengthen immune responses and an excellent record of safety, alum lacks the capability to mediate cell-mediated immunity (4). In contrast, squalene-based emulsion adjuvants, such as MF59 and AS03, can elicit more balanced immunity, possibly by improving antigen uptake, recruiting immune cells, and promoting the migration of activated APCs (4). MF59 and AS03 have already been used in licensed vaccines, with acceptable tolerability and safety profiles in humans (5, 6). MF59 is an adjuvant consisting of squalene oil, a biodegradable surfactant (Span 85), and polysorbate 80 (PS80) (7), and exerts its adjuvant activity when all components are emulsified. Unlike MF59, AS03 contains α-tocopherol; this adjuvant component induces cytokines, such as interleukin (IL)-6, which may contribute to antibody induction and durability of the humoral immune responses by AS03 (8), although the detailed mechanism of action is still unknown.

There is much current interest in finding optimal vaccine adjuvants, both in terms of new vaccine development against severe acute respiratory syndrome coronavirus 2 (SARS-CoV-2) and to prepare for future pandemics (9). As of 17 January 2023, there were 11 vaccines against SARS-CoV-2 that were approved for use around the world; there were also 176 and 199 vaccine candidates in clinical and preclinical development, respectively (10–12).

S-268019-b is a novel SARS-CoV-2 vaccine developed by Shionogi & Co., Ltd., which is currently in clinical development. It consists of full-length recombinant SARS-CoV-2 spike-protein from Pango lineage A (S-910823) as the antigen and a novel emulsion adjuvant (A-910823; composed of squalene, α-tocopherol, and PS80) (13, 14). There is evidence that S-268019-b elicits more sustained antibody responses compared with the use of BNT162b as a booster dose, even in participants with no systemic adverse events (15). Furthermore, interim data from a phase 2/3 study demonstrated that S-268019-b was safe and showed robust immunogenicity as a booster, supporting its use as a SARS-CoV-2 booster dose (14). Additionally, in preclinical studies, S-268019-b enhanced humoral immune responses and exhibited protective efficacy against SARS-CoV-2 JPN/TY/WK-521 (WK-521, accession no. EPI_ISL_408667) in a non-human primate model (16). Further analysis demonstrated the enhanced neutralization breadth against the SARS-CoV-2 Omicron subvariant by homologous and heterologous booster vaccinations (17). Our recent immunological analysis showed the induction of T follicular helper (Tfh) cells, memory B cells, and memory T cells, which confer the duration and breadth of antigen-specific antibodies, by A-910823 in mice (18). Thus, a line of evidence has demonstrated the magnitude and durability of the immune responses induced by S-268019-b (13, 14, 16–18). However, the mechanism of action and the contribution of each component of A-910823 as a vaccine adjuvant are yet to be elucidated. The aim of the current study was to investigate the mechanism of action of A-910823 in a murine model.

## 2 Materials and methods

### 2.1 A-910823, mRNA-LNP, and empty-LNP preparation

Oil-in-water emulsion adjuvant A-910823, A-910823 with various proportions of squalene and α-tocopherol, and A-910823 at different average diameters were prepared at Shionogi & Co., Ltd. (Osaka, Japan) with a high-pressure homogenization technique as previously described (19, 20), with some modifications, which are summarized in the **Supplementary Methods**. The LNP-formulated mRNA vaccine (LNP-mRNA) was prepared using ionizable Lipid8 and mRNA encoding the full-length SARS-CoV-2 spike protein from the Pango lineage A strain, as previously described (17). Lipid8, distearoylphosphatidylcholine (Nippon Fine Chemical Co., Ltd, Tokyo, Japan), cholesterol (Nippon Fine Chemical Co., Ltd), and 1,2-dimyristoyl-sn-glycerol methoxypolyethylene glycol 2000 (NOF Corporation, Osaka, Japan) were dissolved in ethanol. The lipid solution and mRNA in acetate buffer (pH 4.0) were mixed at a flow ratio of 1:3 (v/v) and a total flow rate of 12 mL/min. The mixture was dialyzed with PBS (pH 7.4) at 4°C, and then passed through a 0.45-μm filter. The LNP preparation was analyzed for particle size and mRNA encapsulation. Empty-LNP (eLNP) was prepared in the same way, using acetate buffer (pH 4.0) without mRNA.

### 2.2 Transmission electron microscopy

To observe physicochemical characteristics of A-910823, 50 µL of the sample diluted 10-fold in phosphate buffered saline (PBS) was pipetted onto a copper grid (TED PELLA, Redding, CA, USA) with formvar film and incubated for 5 min. After removing excess liquid, negative staining was performed with 1% uranyl acetate for 5 min. The grid was observed with JEM-1400 Flash (JEOL, Tokyo, Japan).

### 2.3 Animals

Female Balb/cAJcl mice (6–8 weeks old) were purchased from CLEA Japan, Inc. (Tokyo, Japan). All mouse experiments were performed in accordance with the appropriate laws and guidelines approved by the Institute of Medical Science at the University of Tokyo, Tokyo, Japan (approval no. PA21-46) and the Institutional Animal Care and Use Committee of Shionogi & Co., Ltd (approval no. S19017D and S20073C).

### 2.4 Murine immunization procedures

Modified adjuvants, including those with one or more constituents omitted and those with differing particle sizes (described in the **Supplementary Methods**), were administered IM as two doses (1 µg of S-910823 mixed with or without 50% v/v of each modified adjuvant per dose) at 14-day intervals. In detail, after anesthetization, on day 0 and day 14, Balb/c mice received an IM dose of the following to the femoral muscle: recombinant S-910823 (1 µg) in 50 µL PBS solution (antigen-alone group); recombinant S-910823 (1 µg) in 25 µL PBS solution mixed with A-910823 (25 µL), A-910823 without α-tocopherol (25 µL), A-910823 without squalene (25 µL), A-910823 without α-tocopherol and squalene (25 µL), A-910823 of different particle sizes (25 µL), QS21 (5 µg; Creative Biolabs, Shirley, NY, USA), AddaVax (25 µL; InvivoGen, San Diego, CA, USA), alum (250 µg; InvivoGen) or eLNP (equivalent to lipid content of 5 µg mRNA-LNP); or mRNA-LNP (5 µg) in 50 µL PBS solution.

In a heterologous booster study, Balb/c mice received an IM dose (to the femoral muscle) of 3-µg mRNA-LNP as the first and second immunizations and 3-µg mRNA-LNP or S-910823 (1 µg) mixed with A-910823 (25 µL) as a booster dose, on days 0, 20, and 42, respectively.

### 2.5 Specimen preparation

Mice were euthanized and whole blood, draining lymph nodes, spleens, femurs, tibias, and femoral muscles at the injection site were obtained. Sera were prepared from the blood samples and stored at −80°C until the assays were performed. The spleens were dissociated into cell suspensions using a gentleMACS Dissociator (Miltenyi Biotec, Bergisch Gladbach, Germany). The red blood cells were lysed with Red Blood Cell Lysis Buffer (Roche, Basel, Switzerland), and the splenocytes and bone marrow cells were suspended in Roswell Park Memorial Institute-1640 medium (Nacalai Tesque Inc., Kyoto, Japan) supplemented with 10% fetal bovine serum (Sigma-Aldrich, Burlington, MA, USA). The draining lymph nodes were collected, and single-cell suspensions were prepared using BiomusherII (Nippi Research Institute of Biomatrix, Ibaraki, Japan). Femoral muscle samples were fixed with 10% neutral buffered formalin (Mildform 10N; FUJIFILM Wako Pure Chemical, Osaka, Japan) for 24 hours at room temperature (RT), paraffin-embedded, sectioned, and stained with hematoxylin and eosin (H&E).

### 2.6 Histology

Histopathological changes, such as inflammation and necrosis of muscle fiber, were independently evaluated by two pathologists using the H&E-stained specimens.

### 2.7 Measurement of cytokine levels

The Bio-Plex Pro Mouse Cytokine 23-plex Assay kit (Bio-Rad, CA, USA) was used to measure cytokines in mouse serum following the manufacturer’s instructions.

### 2.8 Measurement of anti-receptor-binding domain-specific antibody titer

To measure the immunogenicity after IM dosing, the antigen-specific total immunoglobulin G (IgG), and IgG1 and IgG2a titers were measured by ELISA. In brief, 96-well, half-area plates (Corning Inc., Corning, NY, USA) were coated with 1 μg/mL of SARS-CoV-2 S-protein receptor-binding domain (RBD) from Pango lineage A, His Tag (multi-angle light scattering verified; ACROBiosystems, Newark, DE, USA) overnight at 4°C. After washing with PBS containing 0.05% Tween 20 (PBS-T) (Nacalai Tesque Inc.), the plates were blocked with 1% bovine serum albumin (Sigma-Aldrich) in PBS-T for 1 hour at RT. Sera were 5-fold serially diluted in the range of 1/100 to 1/7,812,500 with PBS-T containing 0.1% bovine serum albumin. The samples were incubated for approximately 2 hours at RT, followed by incubation with horseradish peroxidase-conjugated anti-mouse IgG, IgG1, or IgG2a antibody (Southern Biotech, Birmingham, AL, USA) for approximately 1 hour at RT. TMB Microwell Peroxidase Substrate (KPL, Gaithersburg, MD, USA) was added to initiate the color reaction for approximately 5 min at RT. Finally, sulfuric acid (Nacalai Tesque Inc.) was added to stop color development, and the optical density was measured at 450 nm using a PowerWave HT microplate reader (BioTek, Winooski, VT, USA).

### 2.9 SARS-CoV-2 neutralization assay

Neutralizing antibody levels in the sera collected at day 28 and day 60 were analyzed against ancestral SARS-CoV-2 WK-521 using VeroE6/TMPRSS2 cells as described previously (16) and summarized in the **Supplementary Methods**.

### 2.10 Flow cytometry

Draining lymph nodes were collected at day 28, day 60, and at 4, 16, 24, and 72 hours post-first immunization to analyze Tfh cells and germinal center B (GCB) cells, and inflammatory cells, respectively. Draining lymph node cells were then washed and incubated with anti-CD16/32 (clone 2.4G2; BD Pharmingen, San Diego, CA, USA), before being stained for viability with the Zombie NIR™ Fixable Viability Kit (BioLegend). The cells were then immunostained with the antibodies outlined in **Supplementary Table 1**, before being examined using the BD LSR II flow cytometer (BD Bioscience, San Jose, CA, USA) or the NovoCyte 3000 flow cytometer (Agilent Technologies, Inc., Santa Clara, CA, USA). Data were analyzed by FlowJo software (Tree Star, Ashland, OR, USA) or NovoExpress 1.4.0 software (Agilent Technologies, Inc.).

### 2.11 Enzyme-linked immunospot assay

Antigen-specific cytokine production was measured using enzyme-linked immunospot (ELISpot) assays, which were performed using ELISpotPLUS kits for mouse interferon (IFN)-γ, and IL-4 (Mabtech, Cincinnati, OH, USA), following the manufacturer’s instructions with slight modifications, which is summarized in the **Supplementary Methods**. The number of antigen-specific antibody-secreting cells (ASCs) was measured using a B-cell ELISpot assay with ELISpot polyvinyl difluoride plates and a mouse IgG ELISpot BASIC Kit (Mabtech), following the manufacturer’s instructions with some modifications (18), as summarized in the **Supplementary Methods**.

### 2.12 Statistical analysis

A one-way analysis of variance followed by Tukey’s multiple comparison test was used for groups of three or more. Statistical analyses were performed using GraphPad Prism 9 (GraphPad Software Inc, San Diego, CA, USA).

## 3 Results

### 3.1 A-910823 induces Tfh cells and humoral immune responses

Although clinical trial data demonstrated the efficacy of S-268019-b in humans, detailed knowledge of the characteristics of the induced immune response is still limited. A comparison between the adaptive immune response profile enhanced by A-910823 and that of other adjuvants was conducted to more fully characterize A-910823. AddaVax, QS21, and alum, which have been widely used in adjuvant research, were used for the comparison, and each dose was set as described in previous reports (21–25). In addition to those adjuvants, eLNP was also evaluated. LNP are novel adjuvant components that can be incorporated into mRNA vaccines. LNP-encapsulated nucleoside-modified mRNA vaccines have shown great efficacy against SARS-CoV-2 (26), and animal experiments have demonstrated that LNP has intrinsic adjuvant activity (27). The dose of eLNP was set as lipid content equivalent to 5 µg mRNA-LNP, the dose at which mRNA-LNP induces an acquired immune response (**Supplementary Figure 1**).

**Figure 1:**
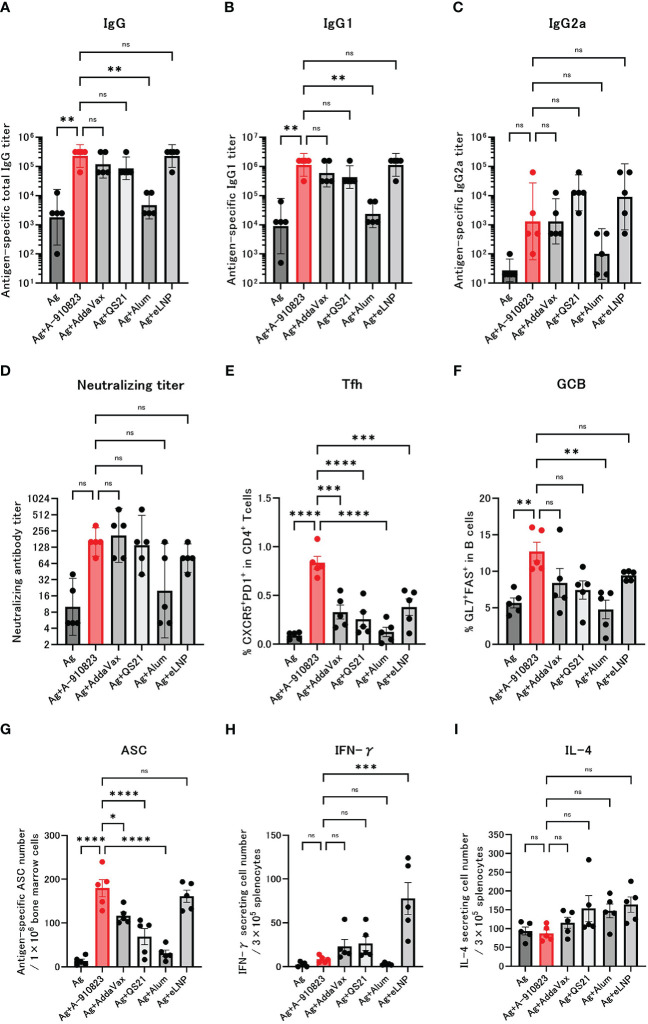
A-910823 induces robust Tfh cells and humoral immune responses. Balb/c mice were injected IM with SARS-CoV-2 S-protein S-910823 (Ag) alone or Ag mixed with each adjuvant on days 0 and 14 (n=5/group). **(A–C)** Antigen-specific total IgG, IgG1, and IgG2a titers in the sera on day 28. Each bar represents the GMT; error bars indicate the 95% confidence interval. Each circle represents the titer in individual mice. **(D)** Serum titers of neutralizing antibodies against SARS-CoV-2 on day 28. Each bar represents the GMT; error bars indicate the 95% confidence interval. Each circle represents the neutralizing antibody titer in individual mice. **(E)** Percentages of Tfh (PD1+CXCR5+) cells in TCRb+CD4+ cells and **(F)** percentages of GCB (FAS+GL7+) cells in CD19+ cells in the draining lymph nodes on day 28. Each bar represents the mean; error bars indicate the standard error of the mean. Each circle represents the percentages of Tfh and GCB cells in individual mice. **(G)** The number of ASCs in bone marrow on day 109 was determined by ELISpot. Each bar represents the mean; error bars indicate the standard error of the mean. Each circle represents the antigen-specific spot-forming cells in individual mice. **(H–I)** The number of cytokine-secreting cells in the spleen of immunized mice on day 28 was determined by ELISpot. Each bar represents the mean number of antigen-specific spot-forming cells; error bars indicate the standard error of the mean. Each circle represents the antigen-specific spot-forming cells in individual mice. Statistical significance was determined using Tukey’s multiple comparison test (ns, P≥0.05, *P<0.05, **P<0.01, ***P<0.001, and ****P<0.0001).

Mice received two IM doses of S-910823 plus A-910823, AddaVax, QS21, alum, or eLNP on days 0 and 14. Anti-RBD total IgG, IgG1, and IgG2a titers were measured 13 days after the second dose on day 28 (**Figures 1A–C**). Compared with the antigen-alone group, the geometric mean titers (GMTs) of total IgG and IgG1 were significantly higher in the A-910823 adjuvant group (shown in red; P=0.0050 and P=0.0017, respectively). Although there was no significant difference in IgG2a GMT between the antigen-alone group and the A-910823 adjuvant groups, the GMT values increased with A-910823. With the exception of alum, there were no significant differences in the GMTs of total IgG, IgG1, and IgG2a induced by A-910823 and other adjuvants.

The GMT of neutralizing antibodies against SARS-CoV-2 WK-521 (Wuhan strain) were also validated (**Figure 1D**). The GMT of the S-910823 only group was 10.0, and the GMT of the S-910823 plus A-910823 group was 160.0. Although no statistically significant differences were identified, the GMT values increased with A-910823. Furthermore, compared with other adjuvants, the S-910823 plus A-910823 group had the second highest GMT value after AddaVax (S-910823 plus AddaVax: 211.1; S-910823 plus QS21: 139.3; S-910823 plus alum: 20.0; and S-910823 plus eLNP: 80.0).

Tfh cells, a helper T cell subset present in germinal centers in LNs, facilitate the generation of GCB, memory B cells, and long-lived plasma cells, which are required for long-term antibody responses (28). The generation of Tfh and GCB in draining lymph nodes was investigated to further understand the effect of A-910823 on those cells. We assumed the Tfh and GCB cell responses were antigen-specific based on the timing of the sampling with respect to vaccination. Tfh and GCB cells were induced at significantly higher levels in the A-910823 adjuvant group compared with the antigen-alone group (P<0.0001 and P=0.0056, respectively; **Figures 1E, F**; **Supplementary Figure 2**). Tfh cell induction was highest in the A-910823-adjuvant group (**Figure 1E**). The mean %GCB in B cells was higher than that in other adjuvants (**Figure 1F**). Furthermore, to quantify long-lived plasma cells (29, 30), we measured the number of SARS-CoV-2 spike-protein-specific ASCs in bone marrow cells on day 109 using an ELISpot assay. ASCs were induced at significantly higher levels in the A-910823 adjuvant group compared with the antigen-alone group (P<0.0001; **Figure 1G**). A-910823 induced significantly higher ASCs than did the other adjuvants, with the exception of eLNP. These results indicate that the adjuvant A-910823 has a high ability to induce humoral immunity. To investigate the cellular immune responses stimulated by the adjuvants, we measured the production of Th1 (IFN-γ) and Th2 (IL-4) cytokines from splenocytes collected on day 28 using an ELISpot assay (**Figures 1H, I**). Although there was no significant difference between the antigen-alone group and A-910823 adjuvant group in the induction of IFN-γ- and IL-4-producing cells, A-910823 slightly enhanced the induction of IFN-γ-producing cells. The highest induction of IFN-γ-producing cells was observed in the eLNP group.

Adjuvants elicit systemic and local immune responses after injection, and those responses are different depending on the characteristics of the adjuvants. We next investigated systemic inflammation and local reaction induced by A-910823. Compared with A-910823, induction of tumor necrosis factor (TNF)-α was particularly high in the eLNP group (**Supplementary Figure 3A**). Compared with A-910823, induction of IL-6 was particularly high in the mRNA-LNP and QS21 groups at 6 hours after injection (**Supplementary Figure 3A**). At 24 hours after injection, S-910823 plus A-910823 induced mild inflammatory cell infiltration in the femoral muscles compared with antigen alone but did not induce necrosis of muscle fiber. Conversely, S-910823 plus QS21 induced not only mild inflammatory cell infiltration but also necrosis of muscle fiber (**Supplementary Figure 3B**), as previously reported in rats (31, 32). Furthermore, at 24 hours after injection, recruitment of neutrophils, eosinophils, and monocytes into the draining lymph nodes was comparable or significantly higher in the A-910823 group compared with other adjuvants (**Supplementary Figure 3C**).

Overall, these results indicate that adjuvant A-910823 enhanced humoral immune responses in mice, following potent Tfh and GCB cell induction, without inducing a strong systemic inflammatory cytokine response.

### 3.2 Heterologous booster dose of S-268019-b after primary immunization with mRNA-LNP induces Tfh cells and humoral immune responses

When given as a booster dose at >6 months after a two-dose BNT162b2 (tozinameran; Pfizer, New York, NY, USA) regimen, both BNT162b2 and S-268019-b elicited anti-spike-protein IgG antibodies and produced T-cell response and neutralizing antibodies in Japanese adults without prior SARS-CoV-2 infection (14). Therefore, we evaluated detailed immunological characteristics, including Tfh and GCB cell induction, using a booster protocol.

**Figure 2:**
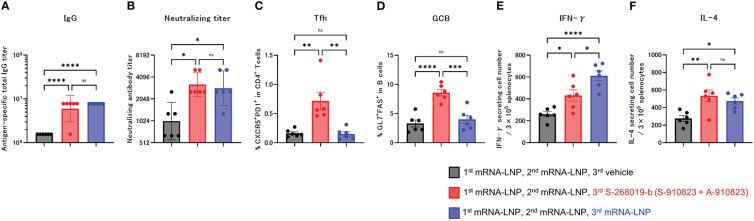
Heterologous booster dose of S-268019-b after primary immunization with mRNA-LNP induces robust Tfh cells and humoral immune responses. Balb/c mice were injected IM with mRNA-LNP as the first and second immunizations, and mRNA-LNP or S-910823 mixed with A-910823 (S-268019-b) as a booster dose, on days 0, 20, and 42, respectively (n=6/group). **(A)** Antigen-specific total IgG titers in the sera on day 60. Each bar represents the GMT; error bars indicate the 95% confidence interval. Each circle represents the titer in individual mice. **(B)** Serum titers of neutralizing antibodies against SARS-CoV-2 on day 60. Each bar represents the GMT; error bars indicate the 95% confidence interval. Each circle represents the neutralizing antibody titer in individual mice. **(C)** Percentages of Tfh (PD1+CXCR5+) cells in TCRb+CD4+ cells and **(D)** percentages of GCB (FAS+GL7+) cells in CD19+ cells in the draining lymph nodes on day 60. Each bar represents the mean; error bars indicate the standard error of the mean. Each circle represents the percentages of Tfh and GCB cells in individual mice. **(E–F)** The number of cytokine-secreting cells in the spleen of immunized mice on day 60 was determined by ELISpot. Each bar represents the mean number of antigen-specific spot-forming cells; error bars indicate the standard error of the mean. Each circle represents the antigen-specific spot-forming cells in individual mice. Statistical significance was determined using Tukey’s multiple comparison test (ns, P≥0.05, *P<0.05, **P<0.01, ***P<0.001, and ****P<0.0001).

Anti-RBD total IgG titers and neutralizing antibodies against SARS-CoV-2 WK-521 were measured 18 days after the booster dose (day 60; **Figures 2A, B** **)**. Total IgG and neutralizing antibody titer of the S-268019-b boost group were higher than in the vehicle group (P<0.0001 and P=0.0197, respectively), and were comparable with those of the mRNA-LNP boost group (P=0.4574 and P=0.9539, respectively). We also investigated whether a booster dose of S-268019-b induced Tfh cells and whether it induced cellular immune responses characteristic of mRNA-LNP. The frequency of Tfh (**Figure 2C**) and GCB (**Figure 2D**) cells in draining lymph nodes in the S-268019-b boost group were higher than that in the vehicle group (P=0.0014 and P<0.0001, respectively) and in the mRNA-LNP boost group (P=0.0012 and P=0.0003, respectively).

In the ELISpot assay, compared with the vehicle group, the S-268019-b-boost group had significantly more IFN-γ- and IL-4-producing cells following splenocyte stimulation (P=0.0260 and P=0.0046, respectively; **Figures 2E, F**). Compared with the mRNA-LNP boost group, the S-268019-b-boost group had a lower number of IFN-γ-producing cells (P=0.0192), but a comparable number of IL-4-producing cells (P=0.6515).

These results indicate that S-268019-b containing A-910823 adjuvant enhanced humoral immune responses with potent Tfh and GCB cell induction, even when given as a booster dose following mRNA vaccine administration.

### 3.3 α-Tocopherol, a component of A-910823, is important for the efficient induction of IgG, Tfh, and GCB cells

**Figure 3:**
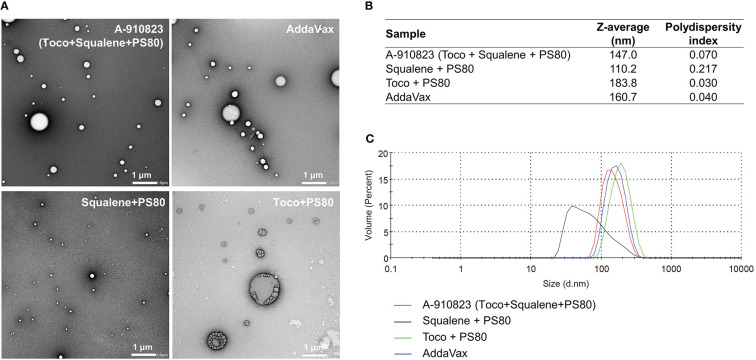
Preparation of A-910823 excluding each component. Physicochemical characterization of A-910823, AddaVax, A-910823 without α-tocopherol (squalene + PS80), and A-910823 without squalene (toco + PS80) determined by **(A)** TEM or **(B-C)** DLS. **(B)** The scale bars represent 1 µm. The hydrodynamic diameter (Z-average size) and polydispersity index of each adjuvant were measured. **(C)** The DLS histograms display the volume-based size distribution of A-910823, AddaVax, A-910823 without α-tocopherol (squalene + PS80), and A-910823 without squalene (toco + PS80).

To identify which components of A-910823 are important for the adjuvant effect (i.e., inducing humoral immune responses and Tfh cells), we prepared modified adjuvants excluding squalene and/or α-tocopherol, and analyzed the physicochemical properties of the modified adjuvants using transmission electron microscopy (TEM) and dynamic light scattering (DLS) techniques. The results revealed that A-910823 formed 147.0-nm particles, which are of comparable size to AddaVax (160.7 nm) (**Figures 3A–C**). α-tocopherol and squalene in A-910823 were found to affect the particle size of A-910823; modified adjuvant lacking α-tocopherol or squalene formed particles with a smaller (110.2 nm) or larger (183.8 nm) diameter than A-910823, respectively (**Figures 3A–C**).

**Figure 4:**
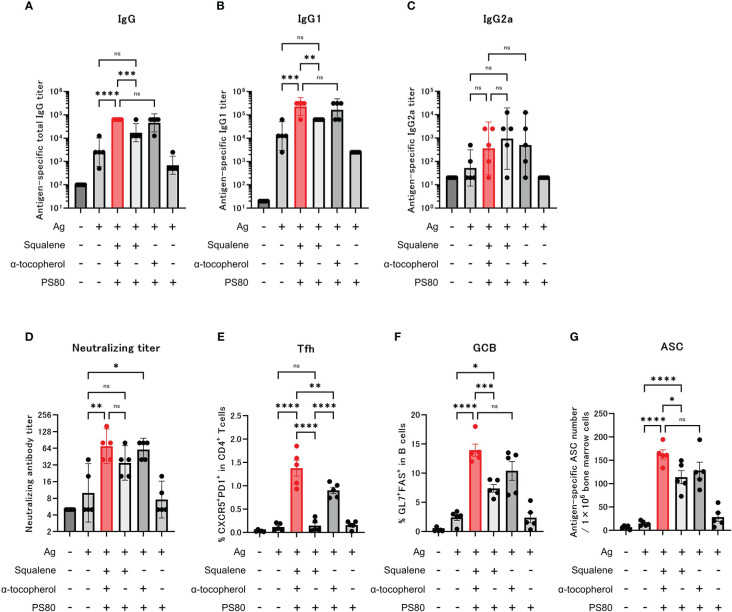
α-tocopherol, a component of A-910823, is important for the efficient induction of IgG, Tfh and GCB cells. Balb/c mice were injected IM with the respective vaccine or vehicle on days 0 and 14 (n=5/group). **(A–C)** Antigen-specific total IgG, IgG1, and IgG2a titers on day 28. Each bar represents the GMT; error bars indicate the 95% confidence interval. Each circle represents the titer in individual mice. **(D)** Serum titers of neutralizing antibodies against SARS-CoV-2 on day 28. Each bar represents the GMT; error bars indicate the 95% confidence interval. Each circle represents the neutralizing antibody titer in individual mice. **(E)** Percentages of Tfh (PD1+CXCR5+) cells in TCRb+CD4+ cells and **(F)** percentages of GCB (FAS+GL7+) cells in CD19+ cells in the draining lymph nodes on day 28. Each bar represents the mean; error bars indicate the standard error of the mean. Each circle represents the percentages of Tfh and GCB cells in individual mice. **(G)** The number of ASCs in bone marrow on day 77 was determined by ELISpot. Each bar represents the mean; error bars indicate the standard error of the mean. Each circle represents the antigen-specific spot-forming cells in individual mice. Statistical significance was determined using Tukey’s multiple comparison test (ns, P≥0.05, *P<0.05, **P<0.01, ***P<0.001, and ****P<0.0001).

Following administration of the modified adjuvants to mice, we observed that compared with the group that was administered modified A-910823 without α-tocopherol, antigen-specific total IgG and IgG1 (**Figures 4A, B**) GMTs measured on day 28 were significantly higher in the A-910823 adjuvant group (P=0.0008 and P=0.0027, respectively). Conversely, A-910823 without squalene induced total IgG and IgG1 GMTs comparable to those induced by A-910823. There was no significant difference in IgG2a GMT between A-910823 and modified A-910823 without α-tocopherol or without squalene (**Figure 4C**).

Neutralizing antibody titers were also validated. The A-910823 adjuvant group and the group that was administered modified A-910823 without squalene each had statistically superior neutralizing antibody titers compared with the S-910823 only group (P=0.0048 and P=0.0497, respectively). However, there was no significant difference between the S-910823 only group and the group that was administered modified A-910823 without α-tocopherol (**Figure 4D**).

Consistent with the results of humoral immune responses, the induction of Tfh and GCB cells by A-910823 on day 28 was highly dependent on α-tocopherol (Tfh, P<0.0001 and GCB, P=0.0006, both A-910823 versus A-910823 without α-tocopherol) (**Figures 4E, F**). Tfh cell induction was significantly reduced by excluding α-tocopherol (P<0.0001) and, to a lesser extent, by excluding squalene (P=0.0046). The induction of ASCs on day 77 was also significantly reduced by excluding α-tocopherol (P=0.0387) (**Figure 4G**). Antigen mixed with PS80 did not enhance the induction of IgG, neutralizing antibody titers, Tfh cells, GCB cells, or ASCs.

Given the variation in particle size of each modified A-910823 demonstrated by the TEM and DLS results, we also evaluated the effect of particle size on humoral immune responses and Tfh and GCB cell induction. We prepared modified A-910823 with the same composition as A-910823 but with different particle sizes (**Supplementary Methods 1.3**; **Supplementary Figures 4A–C**). Measurement of anti-RBD total IgG, and Tfh and GCB cells on day 28 after administration indicated that although particle size influenced Tfh cell induction, it did not affect IgG and GCB cell induction (**Supplementary Figures 4D–F**).

These findings suggest that both the presence of α-tocopherol and the formation of appropriate particle sizes allowed A-910823 to function as a potent Tfh-inducing adjuvant.

### 3.4 Activation of innate immune responses by α-tocopherol

Next, we investigated the effect of α-tocopherol on the innate immune response, in terms of recruitment of inflammatory cells to the draining lymph nodes and induction of serum cytokines and chemokines.

**Figure 5:**
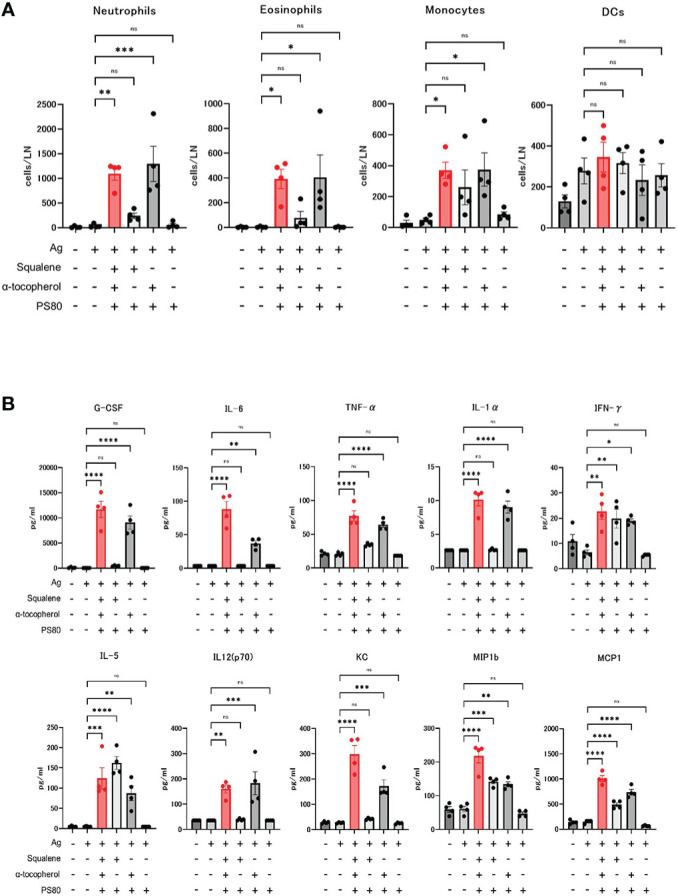
Activation of innate immune responses by α-tocopherol. **(A)** The number of neutrophils, eosinophils, monocytes, and dendritic cells (DCs) in draining lymph nodes at 16 hours post-administration of each adjuvant or vehicle (n=4/group). Each bar represents the mean; error bars indicate the standard error of the mean. Each circle represents the number of inflammatory cells in individual mice. **(B)** Granulocyte-colony stimulating factor (G-CSF), IL-6, TNF-α, IL-1α, IFN-γ, IL-5, IL-12 (p70), KC, macrophage inflammatory protein 1b (MIP1b), and monocyte chemoattractant protein-1 (MCP1) concentrations in sera at 16 hours post-administration of each adjuvant or vehicle (n=4/group). Each bar represents the mean; error bars indicate the standard error of the mean. Each circle represents the cytokine and chemokine concentrations in individual mice. Statistical significance was determined using Tukey’s multiple comparison test (ns, P≥0.05, *P<0.05, **P<0.01, ***P<0.001, and ****P<0.0001).

The absolute number of neutrophils, eosinophils, and monocytes in the draining lymph nodes at 16 hours after the dose of A-910823 mixed with S-910823 was significantly higher compared with S-910823 alone (P=0.0018, P=0.0386, and P=0.0367, respectively). In contrast, these cells were not significantly increased with A-910823 without α-tocopherol. No significant differences were observed in dendritic cells (**Figure 5A**).

Cytokines and chemokines were measured by multiplex analysis, and an A-910823-dependent increase was observed for each molecule (**Figure 5B**). The induction of granulocyte-colony stimulating factor, tumor necrosis factor-α, IL-1α, IL-6, IL-12 (p70), and keratinocyte-derived chemokines (KC) were dependent on α-tocopherol. IFN-γ, IL-5, macrophage inflammatory protein 1b, and monocyte chemoattractant protein-1 were induced by both α-tocopherol and squalene.

These results indicate that recruitment of inflammatory cells to the draining lymph nodes and induction of serum cytokines and chemokines by A-910823 is dependent on α-tocopherol.

## 4 Discussion

A-910823 is a squalene-based adjuvant used in S-268019-b, a novel SARS-CoV-2 vaccine, and the duration and breadth of neutralizing antibodies induced by S-268019-b has been demonstrated in humans and monkeys (14–17). The potent induction of Tfh cells, which enhances the duration and breadth of neutralizing antibodies by generating GCB cells and long-lived plasma cells, has been revealed in our previous research (18); however, the component that contributed to the Tfh cell induction remained unclear. In this study, we provided a novel immunological insight that α-tocopherol is essential for inducing Tfh and high antigen-specific antibody responses.

α-tocopherol, part of the vitamin E family of molecules, is a lipid-soluble antioxidant known to modulate immune function (33) and has been found in higher concentrations in immune cells compared with other cells in the blood (34). Previous studies in humans and animals have shown that vitamin E deficiency, mainly α-tocopherol, impairs both humoral and cellular immunity (35, 36), but these studies have mainly focused on the role of vitamin E as a nutrient. Our study provides a unique perspective by revealing the function of α-tocopherol as an adjuvant. Not only is α-tocopherol a component of A-910823, but it is also a constituent of the squalene-based emulsion adjuvant AS03, which is approved for use in humans (5). Although it is known that α-tocopherol is important for efficient antibody induction (8), this is the first study to demonstrate induction of Tfh by α-tocopherol. In this study, the efficiency of induction of Tfh cells by A-910823 was higher than that of AddaVax, a squalene-based adjuvant without α-tocopherol. Furthermore, although A-910823 is referred to as a “squalene-based adjuvant,” this study newly revealed that its ability to activate innate immune responses, and induce Tfh cells and antibody responses, is more dependent on α-tocopherol than on squalene. In addition to squalene-based adjuvants, there is also evidence that α-tocopherol can enhance humoral immune responses induced by the H1N1 influenza vaccine when added to alum (37). Thus, α-tocopherol may be the main component responsible for the adjuvant effect in AS03 and A-910823, and adding α-tocopherol could lead to the development of new adjuvants with enhanced efficiency in inducing Tfh cells.

The data from this study also indicated that α-tocopherol contributes to the control of A-910823 particle size and is important for the induction of some cytokines and inflammatory cells, and the enhancement of IgG. Like MF59 (7), IM dosing of PS80 did not activate either immune response, suggesting that PS80 is unlikely to damage cells and tissues, or induce damage-associated molecular patterns. However, we also found that rapid inflammatory responses with inflammatory cell infiltration were induced by modified A-910823 lacking squalene in the draining lymph nodes but not by modified A-910823 containing only PS80. Thus, we can infer that α-tocopherol may only induce certain types of stress responses and exert an adjuvant effect.

Previous studies of AS03 have suggested the involvement of the induction of endoplasmic reticulum (ER) stress in adjuvant activity (38). The induction of cytokines such as IL-6 and the induction of Tfh cells in mice after administration of AS03 was inhibited by treatment with 4-phenyl butyric acid, which alleviated ER stress, and knockout of the ER stress sensor kinase IRE1α. These data suggest that the induction of cytokines by A-910823, and even Tfh cell induction, may be mediated by the ER stress-induced axis. Future studies are required to verify why α-tocopherol is important for adjuvant activity, taking into consideration the possibility of ER stress involvement.

The induction of cytokines and chemokines by A-910823 was found to be α-tocopherol-dependent. Of these cytokines, IL-6 is known to be an important cytokine for Tfh cell induction in mice (39), and IL-6-dependent Tfh cell induction has been demonstrated with mRNA-LNP vaccines (27). Therefore, IL-6 may be one of the cytokines involved in Tfh cell induction in A-910823.

Notably, A-910823 with a particle size of approximately 150 nm, which is also used in clinical trials, induced more Tfh cells than those with particle size of 54.3 nm or 385.1 nm. Although A-910823 particle size did not affect IgG antibody titer, it affected Tfh induction, suggesting that α-tocopherol may function both to enhance adjuvant activity through control of A-910823 particle size and through a mechanism unrelated to particle size, such as stress induction. Indeed, emulsion adjuvants of 160 nm are known to induce greater immune cell infiltration into the injection site than those of 20 nm, increasing the number of APCs in the draining lymph nodes and enhancing antigen-specific antibody production (40). In addition, particle size also affects the uptake of adjuvant into the APCs (41). A possible mechanism by which particle size affects adjuvant activity of A-910823 may involve the efficiency of the adjuvant on APCs.

We acknowledge that this study is subject to some limitations; in particular, the effect of these early differences on the duration of the induced immune response remains unknown. Furthermore, unlike previous studies using AS03 (42), we were not able to extensively compare the immune responses elicited by A-910823 with those induced by other adjuvants, including Toll-like receptor agonists. Nonetheless, this study provides a detailed immunological analysis and more detailed mechanism of action of A-910823, which may provide an opportunity to improve Tfh-inducing adjuvants in the future. Previous studies have demonstrated that mRNA vaccines can induce Tfh cells in both humans and mice (25, 43). Thus, we believe that this study on vaccinated mice provides a meaningful approach to elucidate the mechanism of Tfh induction during vaccination in humans. With SARS-CoV-2 infections still spreading worldwide, vaccination remains an important approach to prevent the spread of infection and more people are likely to receive booster doses in the future.

In conclusion, this study found that the novel adjuvant A-910823 is capable of robust Tfh cell induction and humoral immune responses, even when used as a booster dose, and emphasized that the adjuvant activity of A-910823 is driven by α-tocopherol.

## Data availability statement

The original contributions presented in the study are included in the article/**Supplementary Material**. Further inquiries can be directed to the corresponding authors.

## Ethics statement

The animal study was reviewed and approved by the Institute of Medical Science at the University of Tokyo, Tokyo, Japan (approval no. PA21-46) and the Institutional Animal Care and Use Committee of Shionogi & Co., Ltd (approval no. S19017D and S20073C).

## Author contributions

YYo, KK, TH, MO, TN, SI, SO, MN, and KI conceived and designed the study. YYo, TH, MH, YYa, AN, JH, YA, MT, and AH performed experiments and analyzed the data. YYo, MO, and KI drafted the manuscript. All authors contributed to the article and approved the submitted version.
