# Supplementary material for: A-910823, a squalene-based emulsion adjuvant, induces T follicular helper cells and humoral immune responses via α-tocopherol component
Source: Front Immunol. 2023 Feb 20;14:1116238. doi: 10.3389/fimmu.2023.1116238 (PMC9986537; doi:10.3389/fimmu.2023.1116238)
Supplement: Supplementary file 1 [file DataSheet_1.docx]

Supplementary Material

# Supplementary Methods

## Cell Line and Severe Acute Respiratory Syndrome-Coronavirus-2 (SARS-CoV-2)

Transmembrane serine protease 2-expressing VeroE6 (VeroE6/TMPRSS2) cells (1) were obtained from the Japanese Collection of Research Bioresources Cell Bank (Osaka, Japan) and maintained in a culture medium consisting of Dulbecco’s Modified Eagle Medium (Thermo Fisher Scientific, Waltham, MA, USA) containing 10% heat-inactivated fetal bovine serum (Corning Inc., Corning, NY, USA) and 1 mg/mL G418 (Thermo Fisher Scientific).

SARS-CoV-2 JPN/TY/WK-521 (WK-521, accession no. EPI_ISL_408667), which was isolated from the throat swab of a traveler who had returned from Wuhan on January 31, 2020, was provided by the National Institute of Infectious Diseases (Tokyo, Japan) (1).

The WK-521 strain was propagated on VeroE6/TMPRSS2 cells and virus stocks prepared. Titers were calculated using tissue culture infectious dose 50 (TCID50) assays as previously described (2) on VeroE6/TMPRSS2 cells.

## SARS-CoV-2 S-Protein (S-910823) Preparation

The recombinant S-910823 protein was expressed using a baculovirus expression vector system as previously described (3–5). Briefly baculovirus-infected cells were suspended in a buffer containing a nonionic detergent to extract the S-910823 protein. The cell extract was then subjected to centrifugation, and the resulting supernatant was filtered using a depth filter. The S-910823 protein was purified using affinity chromatography and hydrophobic interaction chromatography. Finally, host cell DNA and baculoviruses were removed by Q membrane chromatography.

## A-910823 Preparation

Squalene, α-tocopherol, polysorbate 80 (PS80), and other ingredients of pharmaceutical grade were used for the preparation of A-910823. Squalene, α-tocopherol, and PS80 were mixed in appropriate amounts to produce the adjuvant A-910823 as an oil phase. Phosphate buffer was prepared as a water phase. The oil phase was suspended in the water phase, and then emulsified using high-pressure homogenization. The resulting emulsion and other test samples were analyzed for size and excipient content. The average diameter and polydispersity index were measured by dynamic light scattering (DLS) using Zetasizer nano-ZS ZEN 3600 (Malvern Panalytical, Worcestershire, UK). When measuring DLS, the emulsion was diluted 1:100 in phosphate-buffered saline (PBS). The excipient content was measured using reverse-phase high-performance liquid chromatography (HPLC) (Alliance, Waters, Massachusetts, US). The emulsion was dissolved in 2-propanol, and 10 μL of the solution was then injected into a column (InertSustain C18, 4.6 × 150 mm, 5 μm, GL Sciences, Tokyo, Japan), with the following HPLC conditions: column oven, 35 ºC; flow rate, 1.0 mL/min; UV detection, 220 nm. Methanol and 2-propanol were used for the mobile phase. Modified adjuvants were also prepared, by reducing one or more of the A-910823 constituents (squalene, α-tocopherol, or PS80); these modified adjuvants consisted of smaller volumes compared with that of the unmodified adjuvant. In addition, adjuvants with different particle sizes were prepared by varying the homogenization conditions. Particle sizes were confirmed using by transmission electron microscopy and DLS.

## SARS-CoV-2 Neutralizing Assay

Neutralizing antibody levels in the sera collected at day 28 and day 56 were analyzed against ancestral SARS-CoV-2 WK-521 using VeroE6/TMPRSS2 cells as described previously (5). In brief, heat-inactivated serum samples were serially diluted two-fold (range: 1/5 to 1/5120). Each sample was mixed with an equal volume of viral suspension (2,000 TCID50/mL) and incubated for approximately 1 hour at room temperature for neutralization.

After neutralization, 100 µL of the sample/virus mixtures were dispensed in duplicate into 96-well culture plates. The samples were then incubated with 100 µL of VeroE6/TMPRSS2 cell suspension (3 × 10^4^ cells/well) to obtain a virus titer of 100 TCID50/well. The virus and cell suspension were added to virus control wells, whereas only the cell suspension was added to the cell control wells.

The samples were incubated at 37°C with 5% CO_2_ for 3 days. Cell viability was evaluated using CellTiter-Glo 2.0 (Promega, Madison, WI, USA). After removing 100 μL of supernatant from each well, 100 μL of CellTiter-Glo was added and the samples were incubated at room temperature for approximately 30 min under light shielded conditions. Next, 100 μL of the mixture was transferred to measurement plates and the luminescence intensity was measured using an EnSpire 2300 microplate reader (PerkinElmer, Waltham, MA, USA).

## ELISpot Assay

Antigen-specific cytokine production was measured using ELISpot assays, which were performed using ELISpotPLUS kits for mouse interferon-γ, and interleukin (IL)-4 (Mabtech, Cincinnati, OH, USA), following the manufacturer’s instructions with slight modifications.

In brief, ELISpot plates were washed with PBS and blocked with complete culture medium for at least 1 hour at room temperature. Spike protein overlapping peptides PepTivator SARS-CoV-2 Prot_S, PepTivator® SARS-CoV-2 Prot_S1 and PepTivator® SARS-CoV-2 Prot_S+ (all from Miltenyi Biotec, Bergisch Gladbach, Germany) were dissolved in distilled water and diluted with complete culture medium at a concentration of 180 pmol/mL per peptide. After removing the blocking solution, 100 µL of peptide solution was added to the ELISpot plates. Distilled water in complete culture medium served as a peptide negative (−) control. Next, 100 µL of splenocyte suspension (3 × 10^6^ cells/mL) was added to each well, and the plates were incubated at room temperature for approximately 30 min, followed by incubation at 37°C with 5% CO_2_ overnight. Following five washes with PBS, the samples were incubated with 100 µL of detection antibody solution for 2 hours. Following a further five washes with PBS, the samples were incubated with 100 µL of diluted alkaline phosphatase-conjugated streptavidin solution for at least 1 hour. Finally, the sample was incubated with 100 µL of the chromogen 5-bromo-4-chloro-3-indolyl phosphate/nitro blue tetrazolium, which was used as a chromogen.

The color development was stopped by washing the sample with tap water. The spots were counted using an immunospot S6 UNIVERSAL-V ELISPOT reader (Cellular Technology Limited, Beachwood, OH, USA). The mean spot-forming cell value was calculated for each group. The antigen-specific cytokine production was evaluated by comparing spot-forming cell values of peptide (+) samples with those of peptide (−) control wells.

The number of antigen-specific antibody-secreting cells was measured using a B-cell ELISpot assay. The wells of a 96-well plate were pretreated with 35% ethanol and washed with sterile water, then coated with 5 μg/well SARS-CoV-2 S protein from Pango lineage A (ACROBiosystems, Newark, DE, USA). The plates were incubated overnight at 4°C, washed with PBS, and blocked with complete Roswell Park Memorial Institute 1640 medium for 2 hours at 37°C. The bone marrow cells at day 77 or day 109 were seeded in duplicate (1 × 10^6^ cells/well) and incubated for 2.5 hours at 37°C with 5% CO_2_. The secreted antibodies were detected by incubating with biotinylated polyclonal goat anti-mouse IgG (0.1 μg/well) diluted in 0.5% fetal bovine serum (FBS) in PBS for 2 hours at room temperature. After washing, the samples were incubated with ALP-conjugated streptavidin (Mabtech) diluted in 0.5% FBS/PBS (1:1000) for 1 hour at room temperature. The samples were washed and incubated with the substrate 5-bromo-4-chloro-3-indolyl phosphate/nitro blue tetrazolium (Mabtech; 100 μL/well) for 5 minutes at room temperature. The average number of spot-forming cells per 1 × 10^6^ cells was counted for duplicate wells using an immunospot analyzer (Cellular Technology Limited, Cleveland, OH, USA).

# Supplementary Tables

**Supplementary Table 1 |** List of antibodies.

|  | **Stain** | **Fluorochrome** | **Clone** | **Source** | **Catalogue #** | **Dilution** |
| --- | --- | --- | --- | --- | --- | --- |
| **Tfh/GCB** | Live/dead |  |  | BioLegend | 423106 | 1:200 |
|  | CD95 (Fas) | FITC | SA367H8 | BioLegend | 152606 | 1:200 |
|  | CD19 | PE | 6D5 | BioLegend | 115508 | 1:400 |
|  | CD45R (B220) | PerCP-Cy5.5 | RA3-6B2 | eBioscience | 45-0452-82 | 1:400 |
|  | GL7 | AF647 | GL7 | BioLegend | 144606 | 1:800 |
|  | CD185 (CXCR5) | BV421 | L138D7 | BioLegend | 145512 | 1:200 |
|  | CD4 | BV605 | RM4-5 | BioLegend | 100547 | 1:400 |
|  | TCRβ | BV510 | H57-597 | BD | 563221 | 1:200 |
|  | CD279 (PD-1) | PE-Cy7 | J43 | eBioscience | 25-9985-82 | 1:400 |
| **DC** | Live/dead |  |  | BioLegend | 423106 | 1:200 |
|  | I-A/I-E | BV421 | M5/114.15.2 | BioLegend | 107632 | 1:800 |
|  | CD11c | PE-Dazzle594 | N418 | BioLegend | 117348 | 1:400 |
|  | CD19 | AF700 | 6D5 | BioLegend | 115528 | 1:400 |
| **Neutrophil/ eosinophil/ monocyte** | Live/dead |  |  | BioLegend | 423106 | 1:200 |
|  | CD11b | AF700 | M1/70 | BioLegend | 101222 | 1:400 |
|  | CD11c | BV421 | N418 | BioLegend | 117330 | 1:200 |
|  | Ly6G | BV711 | 1A8 | BioLegend | 127643 | 1:400 |
|  | Ly6C | PE-Cy7 | HK1.4 | BioLegend | 128018 | 1:1000 |
|  | SiglecF | PE-CF594 | E50-2440 | BD | 562757 | 1:400 |

AF, Alexa Fluor; BV, brilliant violet; CD, cluster of differentiation; DC, dendritic cells;
GCB, germinal center B cells; FITC, fluorescein isothiocyanate; LyC, lymphocyte antigen 6 complex locus C; LyG, lymphocyte antigen 6 complex locus G6D; PE, phycoerythrin; TCR, T cell receptor; Tfh, T follicular helper cells.

# Supplementary Figures

**
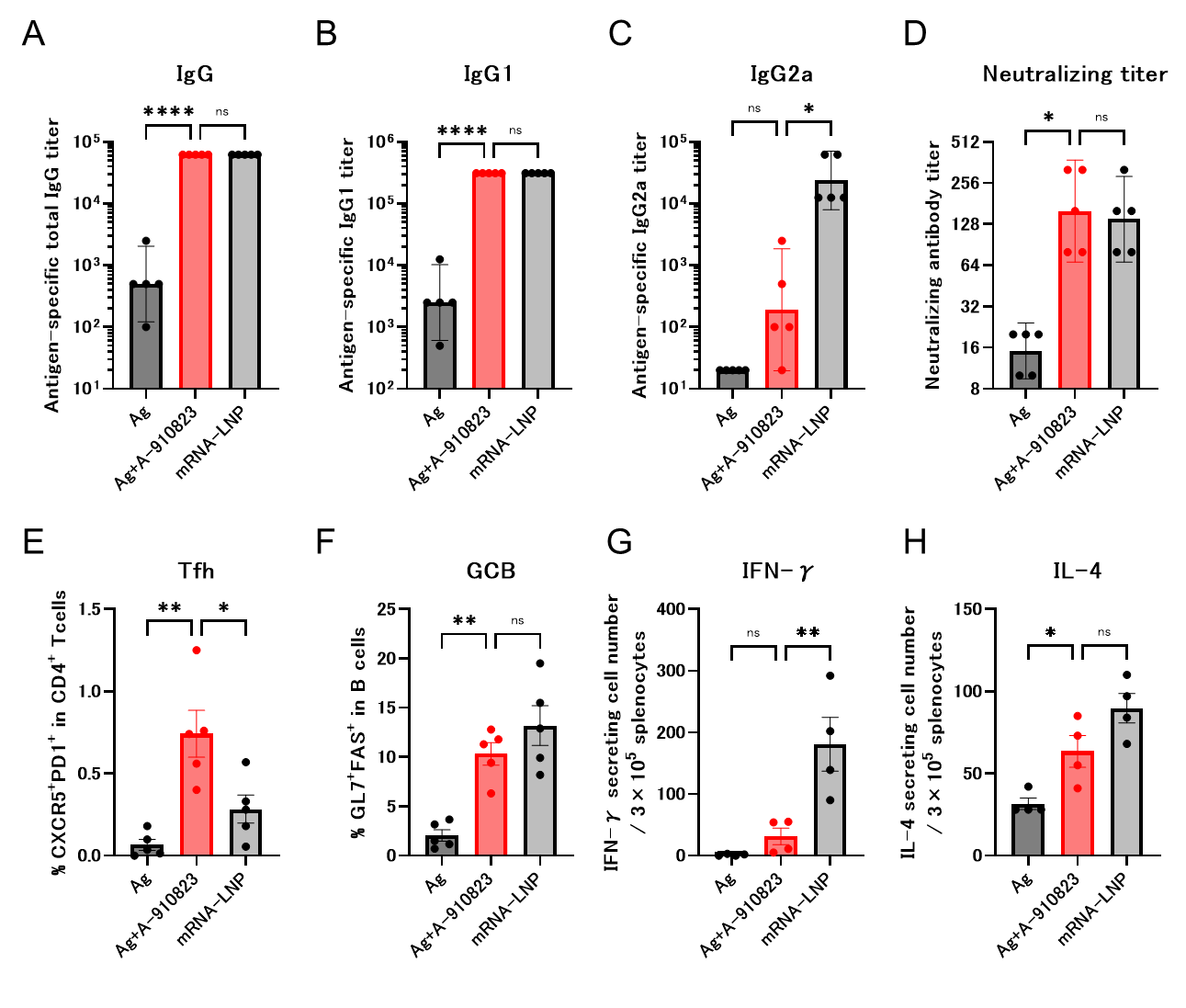
**

**Supplementary Figure 1 |** Induction of acquired immune response by lipid nanoparticle-encapsulated messenger RNA (mRNA-LNP). Balb/c mice were injected intramuscularly with SARS-CoV-2 S-protein S-910823 (Ag) alone, Ag mixed with A-910823, or mRNA-LNP on days 0 and 14 (n=5/group). **(A–C)** Antigen-specific total IgG, IgG1, and IgG2a titers in the sera on day 28. Each bar represents the geometric mean titer; error bars indicate the 95% confidence interval. Each circle represents the titer in individual mice. **(D)** Serum titers of neutralizing antibodies against SARS-CoV-2 on day 28. Each bar represents the geometric mean titer; error bars indicate the 95% confidence interval. Each circle represents the neutralizing antibody titer in individual mice. **(E)** Percentages of T follicular helper (Tfh; PD1+CXCR5+) cells in TCRb+CD4+ cells and **(F)** percentages of germinal center B cells (GCB; FAS+GL7+) cells in CD19+ cells in the draining lymph nodes on day 28. Each bar represents the mean; error bars indicate the standard error of the mean. Each circle represents the percentages of Tfh and GCB cells in individual mice. **(G–H)** The number of cytokine-secreting cells in the spleen of immunized mice on day 28 was determined by ELISpot. Each bar represents the mean number of antigen-specific spot-forming cells; error bars indicate the standard error of the mean. Each circle represents the antigen-specific spot-forming cells in individual mice. Statistical significance was determined using Tukey’s multiple comparison test (ns, P≥0.05, *P<0.05, **P<0.01, and ****P<0.0001).


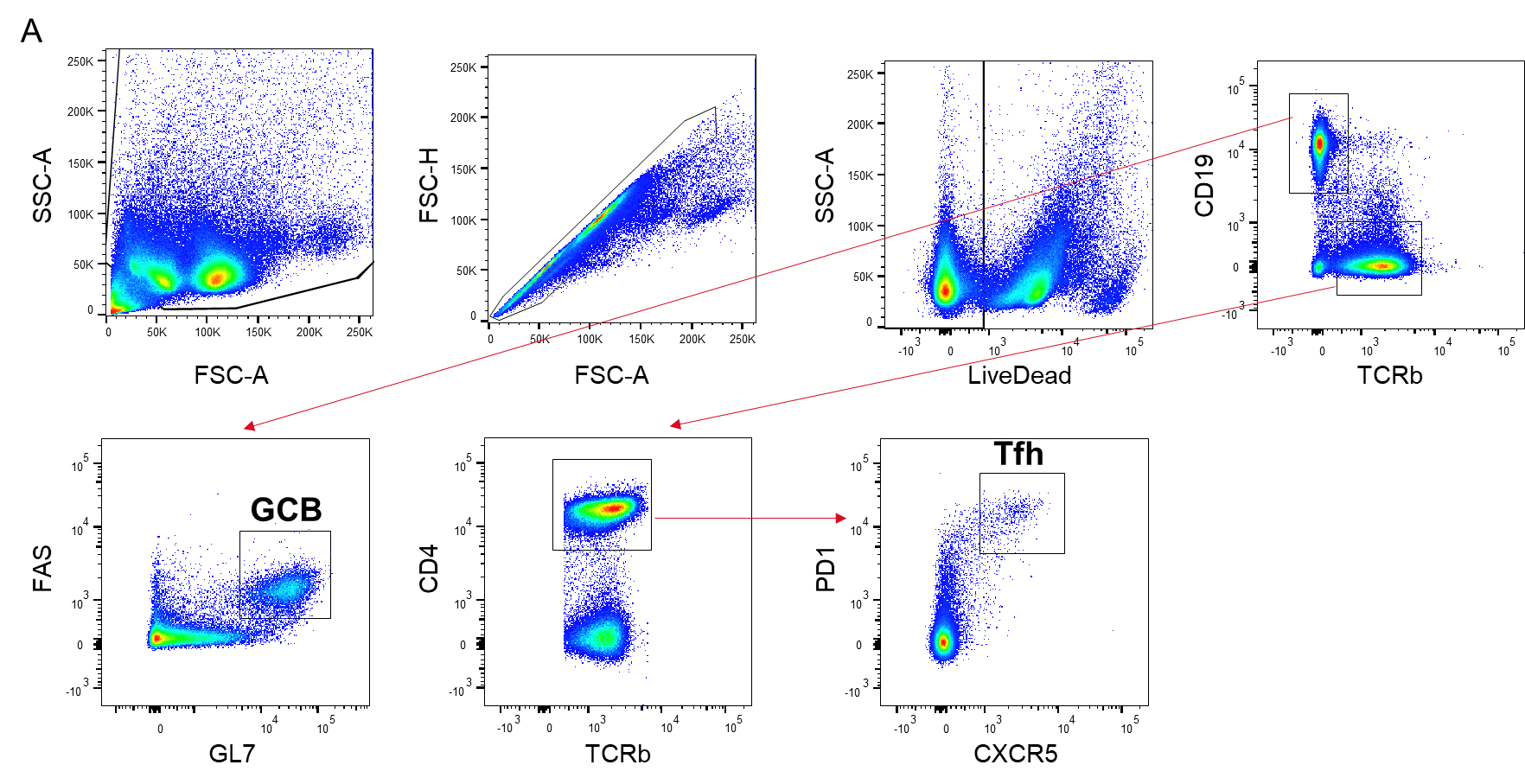


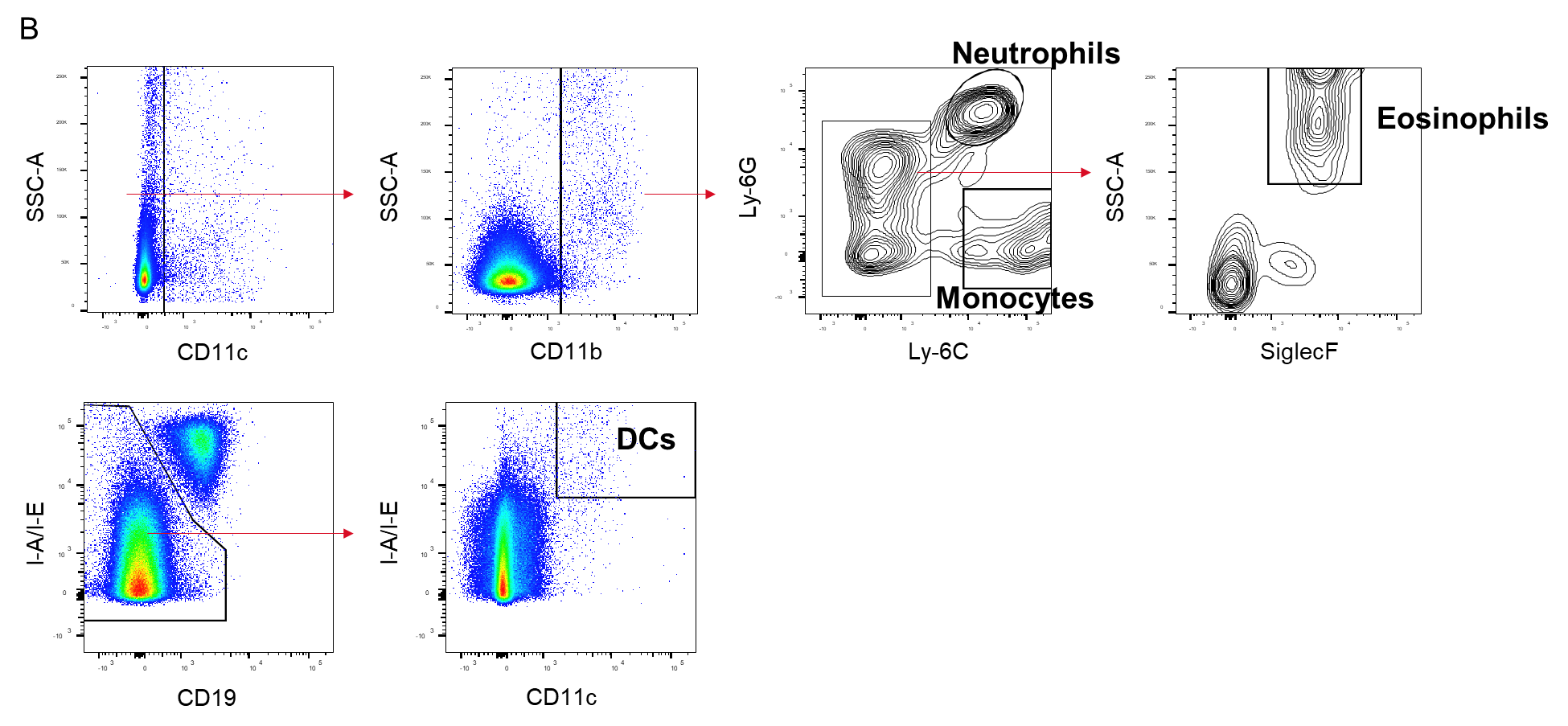


**Supplementary Figure 2 |** Gating strategy. Gating strategy for **(A)** quantification of T follicular helper cells (Tfh) and germinal center B (GCB) cells or **(B)** neutrophils, eosinophils, monocytes, and dendritic cells (DCs). In B, the process of gating live cells is omitted.


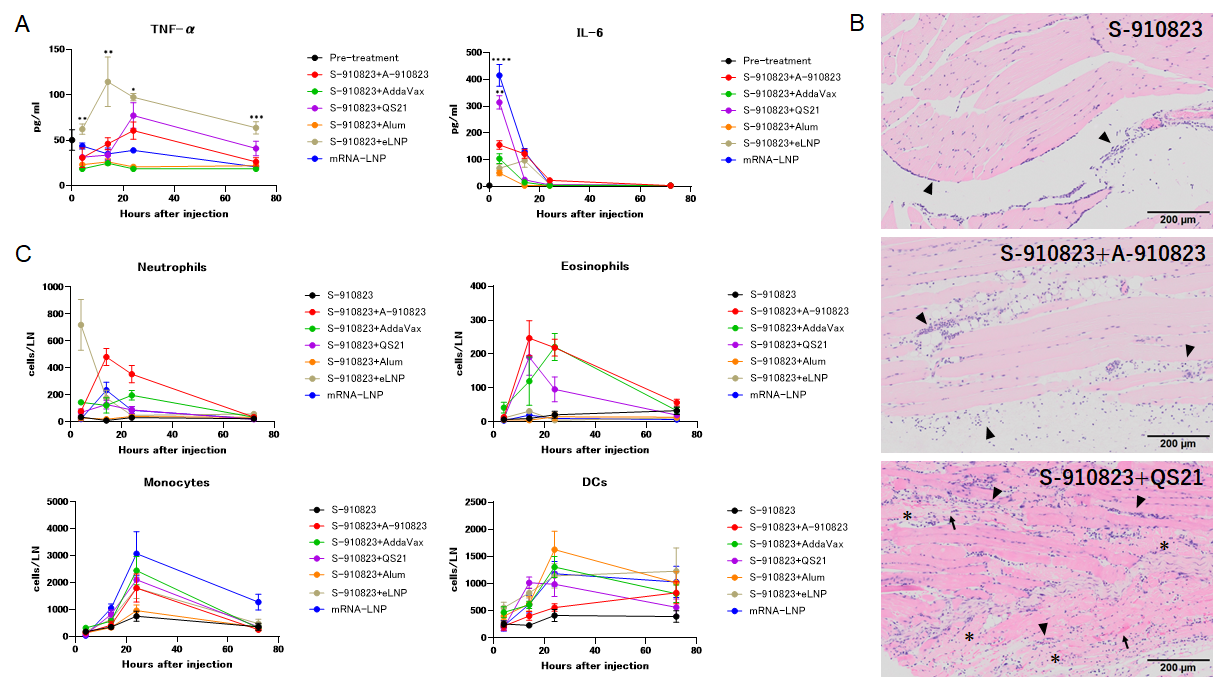


**Supplementary Figure 3 |** Characteristics of each adjuvant. **(A)** Tumor necrosis factor-α and IL-6 concentrations in the sera with the control or the respective adjuvant at pre-administration and at 4, 16, 24, and 72 hours post-administration (n=4/group). Data are represented as mean ± standard error. Statistical significance was determined using Tukey’s multiple comparison test, and statistical differences compared with S-910823 plus A-910823 are indicated (*P<0.05, **P<0.01, and ****P<0.0001). **(B)** Representative hematoxylin and eosin stain images of the femoral muscle for S-910823 alone, S-910823 with A-910823, and S-910823 with QS21 groups at 24 hours post-administration. Annotations are as follows: arrowhead, infiltration of inflammatory cells; arrow, hemorrhage; asterisk, necrosis of muscle fiber. **(C)** The number of neutrophils, eosinophils, monocytes, and dendritic cells in draining lymph nodes with S-910823 alone or the respective adjuvant at 4, 16, 24, and 72 hours post-administration (n=4/group). Data are represented as mean ± standard error.

**
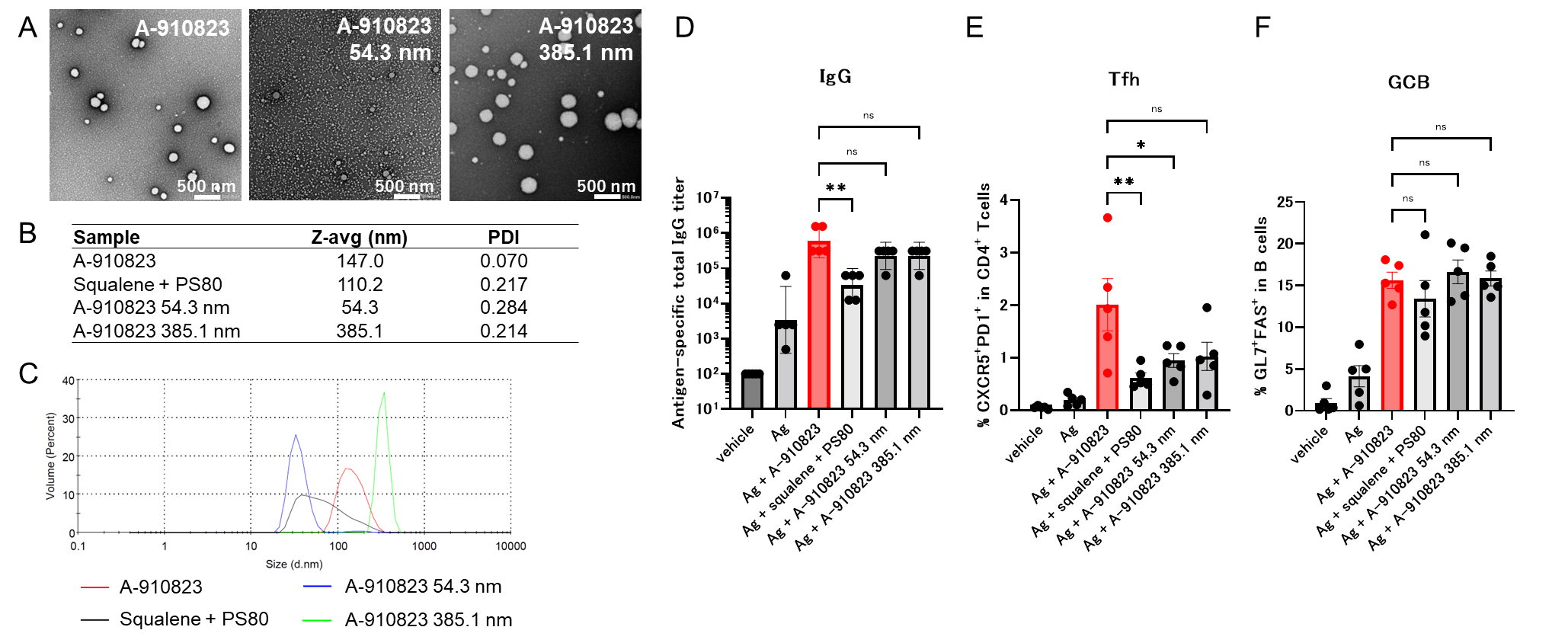
**

**Supplementary Figure 4 |** Relationship between the particle size of A-910823 and adjuvant activity. Physicochemical characterization of A-910823, A-910823 54.3 nm and A-910823 385.1 nm determined by **(A)** transmission electron microscopy or **(B-C)** DLS. **(B)** The scale bars represent 500 nm. The hydrodynamic diameter (Z-average [avg] size) and polydispersity index (PDI) of each adjuvant were measured. **(C)** The DLS histograms display the volume-based size distribution of A-910823, A-910823 without α-tocopherol (squalene + PS80), A-910823 54.3 nm, and A-910823 385.1 nm. **(D)** Balb/c mice (n=5/group) were injected intramuscularly with the respective vaccine or vehicle on days 0 and 14. Antigen-specific total IgG titer in the sera was measured on day 28. Each bar represents the geometric mean titer; error bars indicate the 95% confidence intervals. Each circle represents the titer in individual mice. **(E)** Percentages of T follicular helper (Tfh; PD1^+^CXCR5^+^) cells in TCRb^+^CD4^+^cells and **(F)** percentages of germinal center B (GCB; FAS^+^GL7^+^) cells in CD19^+^cells in the draining lymph nodes on days 28. Each bar represents the mean and error bars indicate the standard error of the mean. Each circle represents the percentages of Tfh and GCB cells in individual mice. Statistical significance was determined using Tukey’s multiple comparison test (ns, P≥0.05, *P<0.05 and **P<0.01).

# References

1. Matsuyama S, Nao N, Shirato K, Kawase M, Saito S, Takayama I, et al. Enhanced isolation of SARS-CoV-2 by TMPRSS2-expressing cells. Proc Natl Acad Sci USA (2020) 117(13):7001-3. doi: 10.1073/pnas.2002589117.
2. Ando Y, Noshi T, Sato K, Ishibashi T, Yoshida Y, Hasegawa T, et al. Pharmacokinetic and pharmacodynamic analysis of baloxavir marboxil, a novel cap-dependent endonuclease inhibitor, in a murine model of influenza virus infection. J Antimicrob Chemother (2021) 76(1):189-98. doi: 10.1093/jac/dkaa393.
3. Contreras-Gómez A, Sánchez-Mirón A, García-Camacho F, Molina-Grima E, Chisti Y. Protein production using the baculovirus-insect cell expression system. Biotechnol Prog (2014) 30(1):1-18. doi: 10.1002/btpr.1842.
4. Cox MM, Hashimoto Y. A fast-track influenza virus vaccine produced in insect cells. J Invertebr Pathol (2011) 107 Suppl:S31-41. doi: 10.1016/j.jip.2011.05.003.
5. Hashimoto M, Nagata N, Homma T, Maeda H, Dohi K, Seki NM, et al. Immunogenicity and protective efficacy of SARS-CoV-2 recombinant S-protein vaccine S-268019-b in cynomolgus monkeys. Vaccine (2022) 40(31):4231-41. doi: 10.1016/j.vaccine.2022.05.081.
